# The generalized Simpson’s entropy is a measure of biodiversity

**DOI:** 10.1371/journal.pone.0173305

**Published:** 2017-03-07

**Authors:** Michael Grabchak, Eric Marcon, Gabriel Lang, Zhiyi Zhang

**Affiliations:** 1 Department of Mathematics and Statistics, University of North Carolina at Charlotte. Charlotte, NC 28223, United States of America; 2 AgroParisTech, UMR EcoFoG, CNRS, CIRAD, INRA, Université des Antilles, Université de Guyane. BP 709, 97310 Kourou, France; 3 UMR 518 Mia, AgroParisTech, INRA, Université Paris-Saclay. F-75015 Paris, France; University of Illinois at Chicago, UNITED STATES

## Abstract

Modern measures of diversity satisfy reasonable axioms, are parameterized to produce diversity profiles, can be expressed as an effective number of species to simplify their interpretation, and come with estimators that allow one to apply them to real-world data. We introduce the generalized Simpson’s entropy as a measure of diversity and investigate its properties. We show that it has many useful features and can be used as a measure of biodiversity. Moreover, unlike most commonly used diversity indices, it has unbiased estimators, which allow for sound estimation of the diversity of poorly sampled, rich communities.

## Introduction

Many indices of biodiversity have been proposed based on different definitions of diversity and different visions of the biological aspects to address [1]. Indeed, measuring diversity requires both a robust theoretical framework [2] and empirical techniques to effectively estimate it [3]. We focus on species-neutral diversity, i.e. the diversity of the distribution of species, ignoring their features. Such measures only make sense when applied to a single taxocene, i.e. a subset of species in the community under study that belong to the same taxon (e.g. butterflies) or, more loosely, to a meaningful group (e.g. trees). Classical measures of this type include richness (the number of species), Shannon’s entropy [4], and Simpson’s index [5].

Since one index is generally insufficient to fully capture the diversity of a community, modern measures of diversity are parameterizable, allowing the user to give more or less relative importance to rare versus frequent species [6]. Further, they can be expressed as an effective number of species [7], which allows for an easy interpretation of their values [8]. Among the most popular indices of this type are HCDT entropy [9–11] (which includes richness, Simpson’s index, and Shannon’s entropy as special cases), Rényi’s entropy [6], and the less-used Hurlbert’s index [12]. These indices can be used to estimate the diversity of a community and then to plot their values against the parameter, which controls the weight of rare species, to obtain a diversity profile [7]. The profiles of two communities can be compared to provide a partial order of their diversity. If the profiles do not cross, one community can be declared to be more diverse than the other [13].

HCDT entropy has many desirable properties [8, 14] but, despite recent progress [15], it cannot be accurately estimated when the communities are insufficiently sampled [16]. Rényi’s entropy is related to HCDT entropy by a straightforward transformation: the natural logarithm of the deformed exponential [14]. Its properties are very similar and, hence, it will not be treated here. Hurlbert’s index has a simple and practical interpretation and can be estimated with no bias, but only up to when its parameter is strictly less than the sample size.

We introduce generalized Simpson’s entropy as a measure of diversity for its particular performance when it is used to estimate the diversity of small samples from hyper-diverse communities. The generalized Simpson’s entropy *ζ*_*r*_ is parameterized: increasing its parameter *r* gives more relative importance to rare species. It has a simple interpretation, specifically, in a species accumulation curve, *ζ*_*r*_ is the probability that the individual sampled at rank *r* + 1 belongs to a new species. We show that *ζ*_*r*_ is a valid measure of diversity, satisfying the axioms established in the literature [2, 6]. We then show how to estimate *ζ*_*r*_ with no bias and how to construct confidence intervals, which can be used to compare the diversities of different communities. After this, we derive a simple formula for the corresponding effective number of species and discuss its estimation. Finally, we compare it to HCDT entropy and Hurlbert’s index on a real-world example of under-sampled tropical forest to illustrate its decisive advantage when applied to this type of data.

## 1 Methods

### 1.1 Generalized Simpson’s entropy

Let *ℓ*_1_, *ℓ*_2_, …, *ℓ_S_* be the species in a community, and let *p*_*s*_ be the proportion of individuals belonging to species *ℓ_s_*. Necessarily, 0 ≤ *p*_*s*_ ≤ 1 and $\sum_{s=1}^{S}p_{s}=1$. We can interpret *p*_*s*_ as the probability of seeing an individual of species *ℓ_s_* when sampling one individual from this community. Generalized Simpson’s entropy is a family of diversity indices defined by

$$\begin{array}{c} \zeta_{r}=\sum_{s=1}^{S}p_{s}{\left(1-p_{s}\right)}^{r},\;\;r=1,2,\ldots . \end{array}$$ (1)

The parameter *r* is called the order of *ζ*_*r*_. Note that, as *r* increases, *ζ*_*r*_ gives more relative weight to rare species than to more common ones. Note further that 0 ≤ *ζ*_*r*_ ≤ 1. In fact, *ζ*_*r*_ is the probability that the (*r* + 1)st observation will be of a species that has not been observed before.

Generalized Simpson’s entropy was introduced as part of a larger class in [17] and was further studied in [18]. The name comes from the fact that 1 − *ζ*_1_ corresponds to Simpson’s index as defined in [5]. A major advantage to working with this family is that there exists an unbiased estimator of *ζ*_*r*_ whenever *r* is strictly less than the sample size. While a similar result holds for Hurlbert’s index, this is not the case with most popular diversity indices including HCDT entropy and Rényi’s entropy, which do not have unbiased estimators. We now turn to the question of when and why generalized Simpson’s entropy is a good measure of diversity.

### 1.2 Axioms for a measure of diversity

Historically, measures of diversity have been defined as functions mapping the proportions *p*_1_, *p*_2_, …, *p*_*S*_ into the real line, and satisfying certain axioms. We write *H*(*p*_1_, *p*_2_, …, *p*_*S*_) to denote a generic function of this type. We begin with three of the most commonly assumed axioms. The first two are from Rényi [6] after Faddeev [19].

**Axiom 1 (Symmetry)** *H*(*p*_1_, *p*_2_, …, *p*_*S*_) *must be a symmetric function of its variables*.

This means that no species can have a particular role in the measure.

**Axiom 2 (Continuity)** *H*(*p*_1_, *p*_2_, …, *p*_*S*_) *must be a continuous function of the vector* (*p*_1_, *p*_2_, …, *p*_*S*_).

This ensures that a small change in probabilities yields a small change in the measure. In particular, two communities differing by a species with a probability very close to 0 have almost the same diversity.

**Axiom 3 (Evenness)** *For a fixed number of species* *S*, *the maximum diversity is achieved when all species probabilities are equal, i.e*.,

$$\begin{array}{c} H\left(p_{1},p_{2},\ldots ,p_{S}\right)\leq H\left(1/S,1/S,\ldots ,1/S\right). \end{array}$$ (2)

This axiom was called evenness by Gregorius [20]. It means that the most diverse community of *S* species is the one where all species have the same proportions.

We will give a more restrictive version of this axiom. Toward this end, following Patil and Taillie [2], we define a *transfer of probability*. This is an operation that consists of taking two species with *p*_*s*_ < *p*_*t*_ and modifying these probabilities to increase *p*_*s*_ by *h* > 0 and decrease *p*_*t*_ by *h*, such that we still have *p*_*s*_ + *h* ≤ *p*_*t*_ − *h*. In other words, some individuals of a more common species are replaced by ones of a less common species, but in such a way that the order of the two species does not change.

**Axiom 4 (Principle of transfers)** *Any transfer of probability must increase diversity*.

The principle of transfers comes from the literature of inequality [21]. It is clear that this axiom is stronger than the axiom of evenness: if any transfer increases diversity, then, necessarily, the maximum value is reached when no more transfer is possible, i.e. when all proportions are equal.

Generalized Simpson’s entropy belongs to an important class of diversity indices, which are called trace-form entropies in statistical physics and dichotomous diversity indices in [2]. This class consists of indices of the form $H\left(p_{1},p_{2},\ldots ,p_{S}\right)=\sum_{s=1}^{S}p_{s}I\left(p_{s}\right)$, where *I*(*p*) is called the information function. Indices of this type were studied extensively in [2] and [20]. *I*(*p*) defines the amount of information [4], or uncertainty [6], or surprise [22]. All of these terms can be taken as synonyms; they get at the idea that *I*(*p*) measures the rarity of individuals from a species with proportion *p* [2]. This discussion leads to the following axiom.

**Axiom 5 (Decreasing information)** *I*(*p*) *must be a decreasing function of* *p* *on the interval* (0, 1] *and* *I*(1) = 0.

This can be interpreted to mean that observing an individual from an abundant species brings less information than observing one from a rare species, and if an individual is observed from a species that has probability 1, then this observation brings no information at all.

Patil and Taillie [2] showed that Axiom 5 ensures that adding a new species increases diversity. They also showed that both the principle of transfers and the axiom of decreasing information are satisfied if the function *g*(*p*) = *pI*(*p*) is concave on the interval [0, 1]. However, for generalized Simpson’s entropy,

$$\begin{array}{c} g\left(p\right)=p{\left(1-p\right)}^{r},\;\;p\in \left[0,1\right] \end{array}$$ (3)

is not a concave function of *p* if *r* > 1. In fact, for *r* > 1 generalized Simpson’s entropy does not satisfy the principle of transfers. For this reason Gregorius [20], in a study of many different entropies, did not retain it. However, we will show that generalized Simpson’s entropies satisfy a weaker version of the principle of transfers, and are, nevertheless, useful measures of diversity.

### 1.3 The generalized Simpson’s entropy is a measure of diversity

It is easy to see that generalized Simpson’s entropy always satisfies Axioms 1, 2 and 5, but, as we have discussed, it does not satisfy Axiom 4. However, we will show that it satisfies a weak version of it and that it satisfies Axiom 3 for a limited, but wide range of orders *r*.

**Axiom 6 (Weak principle of transfers)** *Any transfer of probability must increase diversity as long as the sum of the probabilities of the concerned species is below a certain threshold, i.e., the principle of transfers holds so long as*

$$\begin{array}{c} p_{s}+p_{t}\leq T\;for\;some\;0<T\leq 1. \end{array}$$ (4)

We now give our results about the properties of generalized Simpson’s entropy. The proofs are in S1 Appendix.

**Proposition 1** *Generalized Simpson’s entropy of order* *r* *respects the weak principle of transfers with* $T=\frac{2}{r+1}$.

**Proposition 2** *Generalized Simpson’s entropy of order* *r* *respects the evenness axiom if* *r* ≤ *S* − 1.

In light of Proposition 2, we will limit the order to *r* = 1, 2, …, (*S* − 1). In this case, generalized Simpson’s entropy satisfies Axioms 1–3, and can be regarded as a measure of diversity. Moreover, it satisfies Axiom 5 and the weak principle of transfers up to $T=\frac{2}{r+1}\geq \frac{2}{S}$. Thus, a transfer of probability increases diversity, except between very abundant species.

### 1.4 Estimation

In practice, the proportions, (*p*_1_, *p*_2_, …, *p*_*S*_), are unknown and, hence, the value of generalized Simpson’s entropy as well as any other diversity index is unknown and can only be estimated from data. For this purpose, assume that we have a random sample of *n* individuals from a given community. The assumption that we have a random sample, i.e. that the observations are independent and identically distributed, may be unrealistic in some situations. However, most estimators rely on this assumption, and appropriate sampling design is the simplest solution to obtain independent and identically distributed data. See [23] for a review of these issues in the context of forestry. In principle, the assumption of a random sample implies that either the population is infinite, or that the sampling is done with replacement. In practice, the population is finite and sampling in ecological studies is usually performed without replacement. However, when the sample size is much smaller than the population, the dependence introduced by sampling from a finite population without replacement is negligible and can be ignored.

Let *n*_*s*_ be the number of individuals sampled from species *ℓ_s_*, and note that $n=\sum_{s=1}^{S}n_{s}$. We can estimate *p*_*s*_ by ${\hat{p}}_{s}=n_{s}/n$. A naive estimator of *ζ*_*r*_ is given by the so-called “plug-in” estimator $\sum_{s=1}^{S}{\hat{p}}_{s}{\left(1-{\hat{p}}_{s}\right)}^{r}$. Unfortunately, this may have quite a bit of bias. However, for 1 ≤ *r* ≤ (*n* − 1), an unbiased estimator of *ζ*_*r*_ exists and is given by

$$\begin{array}{c} Z_{r}=\frac{n^{r+1}\left[n-r-1\right]!}{n!}\sum_{s=1}^{S}{\hat{p}}_{s}\prod_{j=0}^{r-1}\left(1-{\hat{p}}_{s}-\frac{j}{n}\right), \end{array}$$ (5)

see [17]. There it is shown that *Z*_*r*_ is a uniformly minimum variance unbiased estimator (umvue) for *ζ*_*r*_ when 1 ≤ *r* ≤ (*n* − 1).

Note that the sum in Eq (5) ranges over all of the species in the community. This may appear impractical since we generally do not know the value of *S*. However, for any species *ℓ_s_* that is not observed in our sample, we have ${\hat{p}}_{s}=0$, and we do not need to include it in the sum. Assume that we have observed *K* ≤ *S* different species in the sample and that these species are $\ell_{1}^{\prime },\ell_{2}^{\prime },\ldots ,\ell_{K}^{\prime }$. For each *s* = 1, 2, …, *K*, let $n_{s}^{\prime }$ be the number of individuals from species $\ell_{s}^{\prime }$ sampled, and let ${\hat{p}}_{s}^{\prime }=n_{s}^{\prime }/n$ be the estimated proportion of species $\ell_{s}^{\prime }$. In this case we can write

$$\begin{array}{c} Z_{r}=\frac{n^{r+1}\left[n-r-1\right]!}{n!}\sum_{s=1}^{K}{\hat{p}}_{s}^{\prime }\prod_{j=0}^{r-1}\left(1-{\hat{p}}_{s}^{\prime }-\frac{j}{n}\right). \end{array}$$ (6)

With a few simple algebraic steps, we can rewrite this in the form

$$\begin{array}{c} Z_{r}=\sum_{s=1}^{K}{\hat{p}}_{s}^{\prime }\prod_{j=1}^{r}\left(1-\frac{n_{s}^{\prime }-1}{n-j}\right), \end{array}$$ (7)

which we have found to be more tractable for computational purposes.

In [17] and [18] it is shown that *Z*_*r*_ is consistent and asymptotically normal. These facts can be used to construct asymptotic confidence intervals. First, define the (*K* − 1) × (*K* − 1) dimensional matrix given by

$$\begin{array}{c} \hat{\Sigma }=\left(\begin{array}{cccc} {\hat{p}}_{1}^{\prime }\left(1-{\hat{p}}_{1}^{\prime }\right) & -{\hat{p}}_{1}^{\prime }{\hat{p}}_{2}^{\prime } & \cdots & -{\hat{p}}_{1}^{\prime }{\hat{p}}_{K-1}^{\prime }\\ -{\hat{p}}_{2}^{\prime }{\hat{p}}_{1}^{\prime } & {\hat{p}}_{2}^{\prime }\left(1-{\hat{p}}_{2}^{\prime }\right) & \cdots & -{\hat{p}}_{2}^{\prime }{\hat{p}}_{K-1}^{\prime }\\ \cdots & \cdots & \cdots & \cdots \\ -{\hat{p}}_{K-1}^{\prime }{\hat{p}}_{1}^{\prime } & -{\hat{p}}_{K-1}^{\prime }p_{2}^{\prime } & \cdots & {\hat{p}}_{K-1}^{\prime }\left(1-{\hat{p}}_{K-1}^{\prime }\right) \end{array}\right) \end{array}$$ (8)

and the (*K* − 1) dimensional column vector ${\hat{h}}_{r}$, where for each *j* = 1, …, (*K* − 1) the *j*th component of ${\hat{h}}_{r}$ is given by

$$\begin{array}{c} {\left(1-{\hat{p}}_{j}^{\prime }\right)}^{r}+r{\hat{p}}_{j}^{\prime }{\left(1-{\hat{p}}_{j}^{\prime }\right)}^{r-1}-{\left(1-{\hat{p}}_{K}^{\prime }\right)}^{r}-r{\hat{p}}_{K}^{\prime }{\left(1-{\hat{p}}_{K}^{\prime }\right)}^{r-1}. \end{array}$$ (9)

When there exists at least one *s* with *p*_*s*_ ≠ 1/*S* (i.e. we do not have a uniform distribution) then an asymptotic (1 − *α*)100% confidence interval for *ζ*_*r*_ is given by

$$\begin{array}{c} Z_{r}\pm z_{\alpha /2}\frac{{\hat{\sigma }}_{r}}{\sqrt{n}}, \end{array}$$ (10)

where

$$\begin{array}{c} {\hat{\sigma }}_{r}=\sqrt{{\hat{h}}_{r}^{T}\hat{\Sigma }{\hat{h}}_{r}} \end{array}$$ (11)

is the estimated standard deviation, ${\hat{h}}_{r}^{T}$ is the transpose of ${\hat{h}}_{r}$, and *z*_*α*/2_ is a number satisfying *P*(*Z* > *z*_*α*/2_) = *α*/2 where *Z* ∼ *N*(0, 1) is a standard normal random variable. Methods for evaluating *Z*_*r*_ and ${\hat{\sigma }}_{r}$ are available in the package *EntropyEstimation* [24] for R [25]. For details about the confidence interval see S1 Appendix.

### 1.5 Comparing distributions

In many situations it is important not only to estimate the diversity of one community, but to compare the diversities of two different communities. Toward this end, we discuss the construction of confidence intervals for the difference between the generalized Simpson’s entropies of two communities.

Fix an order *r* and let $\zeta_{r}^{\left(1\right)}$ and $\zeta_{r}^{\left(2\right)}$ be the generalized Simpson’s entropies of the first and second community respectively. To estimate these, assume that we have a random sample of size *n*_1_ from the first community and a random sample of size *n*_2_ from the second community. Assume further that these two samples are independent of each other and that *r* ≤ (min{*n*_1_, *n*_2_} − 1), where min{*n*_1_, *n*_2_} is the minimum of *n*_1_ and *n*_2_. If both communities satisfy the conditions given in Section 1.4, an asymptotic (1 − *α*)100% confidence interval for the difference $\zeta_{r}^{\left(1\right)}-\zeta_{r}^{\left(2\right)}$ is given by

$$\begin{array}{c} \left[Z_{r}^{\left(1\right)}-Z_{r}^{\left(2\right)}\right]\pm z_{\alpha /2}\sqrt{\frac{{\left[{\hat{\sigma }}_{r}^{\left(1\right)}\right]}^{2}}{n_{1}}+\frac{{\left[{\hat{\sigma }}_{r}^{\left(2\right)}\right]}^{2}}{n_{2}}}, \end{array}$$ (12)

where $Z_{r}^{\left(1\right)}$ and $Z_{r}^{\left(2\right)}$ are the estimates of $\zeta_{r}^{\left(1\right)}$ and $\zeta_{r}^{\left(2\right)}$ and ${\hat{\sigma }}_{r}^{\left(1\right)}$ and ${\hat{\sigma }}_{r}^{\left(2\right)}$ are the estimated standard deviations as in Eq (11).

In practice, it is often not enough to look at only one diversity index. For this reason we may want to look at an entire profile of generalized Simpson’s entropies. This can be done as follows. Fix any positive integer *v* ≤ (min{*n*_1_, *n*_2_} − 1). In order for *ζ*_*v*_ to be a reasonable diversity estimator, we also require *v* ≤ (*S* − 1). For each *r* = 1, 2, …, *v* we can estimate $Z_{r}^{\left(1\right)}$, $Z_{r}^{\left(2\right)}$, and the corresponding confidence interval. Looking at these for all values of *r* gives a pointwise confidence envelope. We can now see if the two communities have statistically significant differences in the amount of diversity by seeing if zero is in the envelope or not. If it is generally in the envelope then the differences are not significant, and if it is generally outside of the envelope then the differences are significant.

### 1.6 Effective number of species

The effective number of species [7] is the number of equiprobable species that would yield the same diversity as a given distribution [26]. It is a measure of diversity *sensu sticto* [8]. We will write *entropy* for *ζ*_*r*_ and *diversity* for its effective number, which we denote by ^*r*^*D*^*ζ*^. To derive ^*r*^*D*^*ζ*^ we assume

$$\zeta_{r}=\sum_{s=1}^{{}_{}^{r}\;D_{}^{\zeta }}\frac{1}{{}_{}^{r}\;D_{}^{\zeta }}{\left(1-\frac{1}{{}_{}^{r}\;D_{}^{\zeta }}\right)}^{r},$$ (13)

and then simple algebra yields

$${}_{}^{r}\;D_{}^{\zeta }=\frac{1}{1-\zeta_{r}^{\frac{1}{r}}}.$$ (14)

Note that Eq (13) assumes that ^*r*^*D*^*ζ*^ is an integer, while in Eq (14) it is generally not an integer. This is not an issue because Eq (13) is just a formalism used to derive Eq (14). A more developed argumentation can be found in Appendix B of [20].

Since the function *f*(*t*) = 1/(1 − *t*^1/*r*^), *t* ∈ [0, 1] is monotonically increasing, we can transform confidence intervals for *ζ*_*r*_ into confidence intervals for ^*r*^*D*^*ζ*^ as follows. If (*L*, *U*) is a (1 − *α*)100% confidence interval for *ζ*_*r*_ then (*f*(*L*), *f*(*U*)) is a (1 − *α*)100% confidence interval for ^*r*^*D*^*ζ*^. It is important to note that any inference based on such confidence intervals for ^*r*^*D*^*ζ*^ is equivalent to inference based on the original confidence interval for *ζ*_*r*_.

## 2 Example data and results

In this section we apply our methodology to estimate and compare the diversities of two 1-ha plots (#6 and #18) of tropical forest in the experimental forest of Paracou, French Guiana [27]. Respectively 641 and 483 trees with diameter at breast height over 10 cm were inventoried. The data is available in the *entropart* package for R.

**Fig 1:**
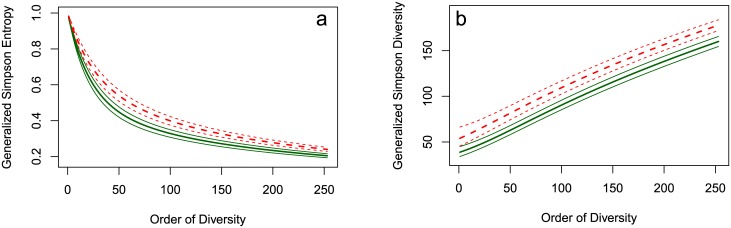
Generalized Simpson’s entropy and diversity profiles. (a) entropy and (b) diversity profiles of Paracou plots 6 (solid, green lines) and 18 (dotted, red lines). The bold lines represent the estimated values, surrounded by their 95% confidence envelopes.

In the data, we observe 147 and 149 species from plots 6 and 18 respectively. However, species may not have been sampled and we must adjust these values. Jackknives tend to be good estimators of richness, see [28]. We use a jackknife of order 2 for plot 6 and one of order 3 for plot 18: the choice of the optimal order follows both [28] and [29]. The estimated richness is, respectively, 254 and 309 species. For this reason we estimate generalized Simpson’s entropy up to order *r* = 253. This, along with a 95% confidence envelope is given in Fig 1a.

The generalized Simpson’s diversity profiles along with a 95% confidence envelope are given in Fig 1b. These give more intuitive information since they represent the effective numbers of species. Their values at *r* = 1 are given, respectively, by 39 and 46 species. Increasing values of *r* give more importance to rare species, which leads to the increase in the effective number of species seen in the graph.

**Fig 2:**
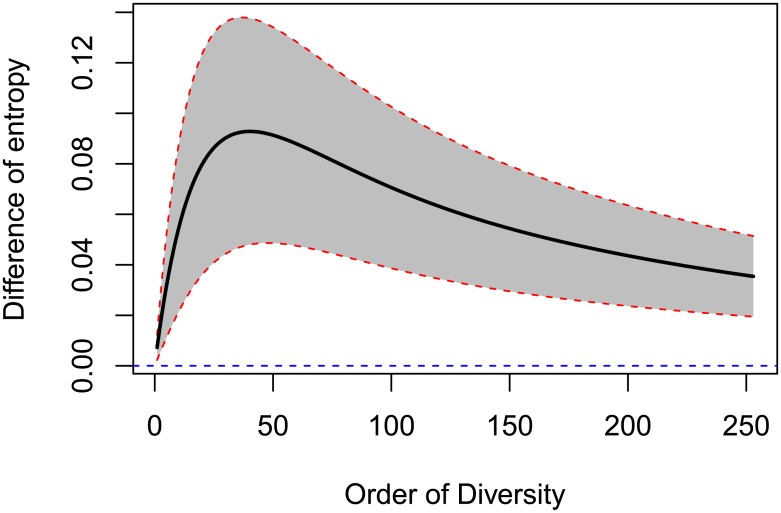
Difference between the generalized Simpson’s entropy of plots 6 and 18 with their 95% confidence envelope. The horizontal dotted line represents the null hypothesis of identical diversity. Since it is always outside of the confidence envelope, identical diversity is rejected.

Plot 18 is clearly more diverse than plot 6, with a fairly stable difference of between 15 and 19 effective species. In Fig 2 the difference between the entropies is plotted with its 95% confidence envelope to test it against the null hypothesis of zero difference. Since zero is never in this envelope, we conclude that plot 18 is significantly more diverse than plot 6.

## 3 Discussion

### 3.1 Interpretation

Generalized Simpson’s entropy of order *r* can be interpreted as the average information brought by the observation of an individual. Its information function *I*(*p*) = (1 − *p*)^*r*^ represents the probability of not observing a single individual of a species with proportion *p* in a sample of size *r*. Thus *I* is an intuitive measure of rarity.

Olszewski [30] (see also [31]) interpreted *ζ*_*r*_ as the probability that the individual sampled at rank (*r* + 1) belongs to a previously unobserved species in a species accumulation curve, i.e. the slope of the curve at rank (*r* + 1). A related interpretation is as follows. If *X* is the number of species observed exactly once in a sample of size (*r* + 1), then *ζ*_*r*_ = E[*X*]/(*r* + 1).

These interpretations are not limited to orders *r* < *S*. However, when *r* ≥ *S*, *ζ*_*r*_ is no longer a reasonable measure of diversity. In particular, in this case, it may not be maximized at the uniform distribution, which could lead the effective number of species, ^*r*^*D*^*ζ*^, to be greater than the actual number of species.

### 3.2 HCDT entropy

**Fig 3:**
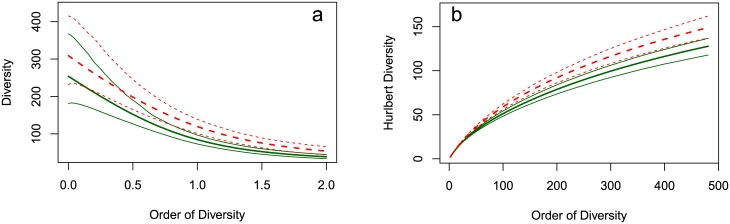
(a) HCDT and (b) Hurlbert’s diversity profiles of Paracou plots 6 (solid, green lines) and 18 (dotted, red lines). The bold lines represent the estimated values, surrounded by their 95% confidence envelope (obtained by 1000 bootstraps).

In this section we compare our results to those based on the more standard HCDT entropy, which is given by

$$\begin{array}{c} {}_{}^{q}\;T_{}^{}=\frac{\sum_{s=1}^{S}p_{s}^{q}-1}{1-q},\;q\geq 0, \end{array}$$ (15)

where, for *q* = 1, this is interpreted by its limiting value as ${}_{}^{1}\;T_{}^{}=-\sum_{s=1}^{S}p_{s}\log p_{s}$. The effective number of species for HCDT entropy was derived in [7]. It is given by

$$\begin{array}{c} {}_{}^{q}\;D_{}^{T}={\left(\sum_{s=1}^{S}p_{s}^{q}\right)}^{1/(1-q)},\;q\geq 0, \end{array}$$ (16)

where, for *q* = 1, this is interpreted by its limiting value as ${}_{}^{q}\;D_{}^{T}=e^{{}_{}^{1}\;T_{}^{}}$. We call this quantity HCDT diversity, although in the literature it is often called Hill’s diversity number. For our data, plots of ^*q*^*D*^*T*^ for *q* ∈ [0, 2] along with a 95% confidence envelope are given in Fig 3a. Here ^*q*^*D*^*T*^ was estimated using the jackknife-unveiled estimator of [16] and the confidence envelope was estimated using bootstrap.

It is easy to see that the importance of rare species increases for HCDT entropy as *q* *decreases*. For comparison, the importance of rare species for generalized Simpson’s entropy increases as *r* *increases*. Note that ^2^*T* = *ζ*_1_. To see what values of *q* in HCDT entropy correspond to other values of *r* for generalized Simpson’s entropy, we can find when ^*r*^*D*^*ζ*^ = ^*q*^*D*^*T*^. Since we can only use *ζ*_*r*_ up to *r* = *S* − 1 it is of interest to find which value of *q* corresponds to this value. For our data we find that in plot 6 *q* = 0.5 corresponds to *r* = 253 and in plot 18 *q* = 0.55 corresponds to *r* = 308.

The main difficulty in working with HCDT entropy is that its estimators have quite a lot of bias, especially for smaller values of *q* [16]. This is illustrated in Fig 3a, where we see that the confidence intervals of the estimated values of the HCDT diversity of plots 6 and 18 have significant overlap up to *q* = 0.75.

Bias is not an issue with generalized Simpson’s entropy, which can be estimated with no bias, regardless of the sample size (although its precision does depend on the sample size, see Eq (10)). The main issue with generalized Simpson’s entropy is that it can only be considered for orders *r* ≤ *S* − 1, and larger values of *r* correspond to smaller values of *q* for HCDT entropy. In our example, the generalized Simpson’s diversity profile can be compared to the part of the HCDT diversity profile between *q* = 0.5 and *q* = 2. Focusing more on rare species is not possible. HCDT diversity allows that theoretically, but is seriously limited by its estimation issues: the profile has a wide confidence envelope and is not conclusive below *q* = 0.75.

On the whole, generalized Simpson’s entropy allows for a more comprehensive comparison of diversity profiles. If richness were greater, higher orders of generalized Simpson’s diversity could be used and estimated with no bias, while low-order HCDT estimation would get more uncertain [16].

### 3.3 Hurlbert’s diversity

Another measure of diversity, which is related to generalized Simpson’s entropy, was introduced in [12]. It is given by

$$\begin{array}{c} {}_{}^{k}\;H_{}^{}=\sum_{s=1}^{S}\left[1-{\left(1-p_{s}\right)}^{k}\right],\;k=1,2,\ldots , \end{array}$$ (17)

and corresponds to the expected number of species found in a sample of size *k*. It is easily verified that ^2^*H* = 1 + *ζ*_1_, and that the higher the value of *k*, the greater the importance given to rare species. While there is no simple formula for the corresponding effective number of species, an iterative procedure for finding it was developed in [32].

Hurlbert [12] developed an unbiased estimator of ^*k*^*H* for all *k* smaller than the sample size. This is similar to what is needed to estimate generalized Simpson’s entropy, although, generalized Simpson’s entropy also needs *r* < *S* for it to be a measure of diversity. We estimate Hurlbert’s index for the two plots, convert them into effective numbers of species, and use bootstrap to get a 95% confidence envelope. The results are given in Fig 3b. We see that the maximum effective numbers of species are well below those of the generalized Simpson’s diversity. Thus Hurlbert’s diversity finds fewer rare species, making it a less interesting alternative for our purpose.

## 4 Conclusion

Generalized Simpson’s entropy is a measure of diversity respecting the classical axioms when *r* < *S* and has a simple formula to transform it into an effective number of species. It faces several issues that limit its use. Specifically, it only makes sense when applied to a single taxocene and its estimator has nice properties only under the assumption of random sampling. However, these issues are shared with all of the other measures of diversity discussed here and many, if not most, of the ones available in the literature. Further, generalized Simpson’s entropy has a decisive advantage over other such measures: it has an easy-to-calculate uniformly minimum variance unbiased estimator, which is consistent and asymptotically normal. These properties make it a useful tool for estimating diversity and for comparing hyper-diverse, poorly sampled communities. R code to reproduce the examples in the paper, based on the packages *EntropyEstimation* and *entropart* [22], is given in S2 Appendix. All data are available in the *entropart* package.

## Supporting information

S1 Appendix Proofs. (PDF)

S2 Appendix R code. This code allows for the reproduction of all examples and figures in this article.
(PDF)

## References

[1] Ricotta C. Through the jungle of biological diversity. Acta Biotheoretica. 2005;53(1):29–38. 10.1007/s10441-005-7001-6 15906141

[2] Patil GP, Taillie C. Diversity as a concept and its measurement. Journal of the American Statistical Association. 1982;77(379):548–561. 10.2307/2287712

[3] Beck J, Schwanghart W. Comparing measures of species diversity from incomplete inventories: an update. Methods in Ecology and Evolution. 2010;1(1):38–44. 10.1111/j.2041-210X.2009.00003.x

[4] Shannon CE. A Mathematical Theory of Communication. The Bell System Technical Journal. 1948;27:379–423, 623–656. 10.1002/j.1538-7305.1948.tb01338.x

[5] Simpson EH. Measurement of diversity. Nature. 1949;163(4148):688 10.1038/163688a0

[6] Rényi A. On Measures of Entropy and Information. In: Neyman J, editor. 4th Berkeley Symposium on Mathematical Statistics and Probability. vol. 1. Berkeley, USA: University of California Press; 1961. p. 547–561.

[7] Hill MO. Diversity and Evenness: A Unifying Notation and Its Consequences. Ecology. 1973;54(2):427–432. 10.2307/1934352

[8] Jost L. Entropy and diversity. Oikos. 2006;113(2):363–375. 10.1111/j.2006.0030-1299.14714.x

[9] Havrda J, Charvát F. Quantification method of classification processes. Concept of structural a-entropy. Kybernetika. 1967;3(1):30–35.

[10] Daróczy Z. Generalized information functions. Information and Control. 1970;16(1):36–51. 10.1016/S0019-9958(70)80040-7

[11] Tsallis C. Possible generalization of Boltzmann-Gibbs statistics. Journal of Statistical Physics. 1988;52(1):479–487. 10.1007/BF01016429

[12] Hurlbert SH. The Nonconcept of Species Diversity: A Critique and Alternative Parameters. Ecology. 1971;52(4):577–586. 10.2307/1934145 28973811

[13] Tothmeresz B. Comparison of different methods for diversity ordering. Journal of Vegetation Science. 1995;6(2):283–290. 10.2307/3236223

[14] Marcon E, Scotti I, Hérault B, Rossi V, Lang G. Generalization of the Partitioning of Shannon Diversity. Plos One. 2014;9(3):e90289 10.1371/journal.pone.0090289 24603966 PMC3946064

[15] Chao A, Jost L. Estimating diversity and entropy profiles via discovery rates of new species. Methods in Ecology and Evolution. 2015;6(8):873–882. 10.1111/2041-210X.12349

[16] Marcon E. Practical Estimation of Diversity from Abundance Data. HAL. 2015;01212435(version 2).

[17] Zhang Z, Zhou J. Re-parameterization of multinomial distributions and diversity indices. Journal of Statistical Planning and Inference. 2010;140(7):1731–1738. 10.1016/j.jspi.2009.12.023

[18] Zhang Z, Grabchak M. Entropic Representation and Estimation of Diversity Indices. Journal of Nonparametric Statistics. 2016;28(3):563–575. 10.1080/10485252.2016.1190357

[19] Faddeev DK. On the concept of entropy of a finite probabilistic scheme. Uspekhi Mat Nauk. 1956;1(67):227–231.

[20] Gregorius HR. Partitioning of diversity: the “within communities” component. Web Ecology. 2014;14:51–60. 10.5194/we-14-51-2014

[21] Dalton H. The measurement of the inequality of incomes. The Economic Journal. 1920;30(119):348–361. 10.2307/2223525

[22] Marcon E, Hérault B. entropart, an R Package to Partition Diversity. Journal of Statistical Software. 2015;67(8):1–26. 10.18637/jss.v067.i08

[23] Corona P, Franceschi S, Pisani C, Portoghesi L, Mattioli W, Fattorini L. Inference on diversity from forest inventories: a review. Biodiversity and Conservation. 2015;in press.

[24] Cao L, Grabchak M. EntropyEstimation: Estimation of Entropy and Related Quantities; 2014. Available from: http://cran.r-project.org/package=EntropyEstimation.

[25] R Development Core Team. R: A Language and Environment for Statistical Computing; 2016. Available from: http://www.r-project.org.

[26] Gregorius HR. On the concept of effective number. Theoretical population biology. 1991;40(2):269–83. 10.1016/0040-5809(91)90056-L 1788824

[27] Gourlet-Fleury S, Guehl JM, Laroussinie O. Ecology & Management of a Neotropical Rainforest Lessons Drawnfrom Paracou, a Long-Term Experimental Research Site in French Guiana. Paris, France: Elsevier; 2004.

[28] Burnham KP, Overton WS. Robust Estimation of Population Size When Capture Probabilities Vary Among Animals. Ecology. 1979;60(5):927–936. 10.2307/1936861

[29] Brose U, Martinez ND, Williams RJ. Estimating species richness: Sensitivity to sample coverage and insensitivity to spatial patterns. Ecology. 2003;84(9):2364–2377. 10.1890/02-0558

[30] Olszewski TD. A unified mathematical framework for the measurement of richness and evenness within and among multiple communities Oikos. 2004;104(2):377–387.

[31] Chao A, Wang YT, Jost L. Entropy and the species accumulation curve: a novel entropy estimator via discovery rates of new species. Methods in Ecology and Evolution. 2013;4(11):1091–1100. 10.1111/2041-210X.12108

[32] Dauby G, Hardy OJ. Sampled-based estimation of diversity sensu stricto by transforming Hurlbert diversities into effective number of species. Ecography. 2012;35(7):661–672. 10.1111/j.1600-0587.2011.06860.x

