# Supplementary material for: The generalized Simpson’s entropy is a measure of biodiversity
Source: PLoS One. 2017 Mar 7;12(3):e0173305. doi: 10.1371/journal.pone.0173305 (PMC5340404; doi:10.1371/journal.pone.0173305)
Supplement: S1 Appendix — (PDF) [file pone.0173305.s001.pdf]

The Generalized Simpson's Entropy is a Measure of Biodiversity. *PLOS ONE*.
Grabchak, M., Marcon, É., Lang, G. & Zhang, Z.
Corresponding author: Éric Marcon, UMR EcoFoG. (Eric.Marcon@ecofog.gf)

S1 Appendix: Proofs

The proof of Proposition 1 is given first. The gradient and Hessian of the generalized Simpson's entropy are then calculated and the proof of the satisfaction of the evenness axiom is given. Finally, we explain where the confidence intervals come from.

Several of the proofs require understanding the properties of the function $g(p) = p(1-p)^r$ and its first derivative. For $p \in [0,1)$ the first two derivatives of g are

$$g'(p) = (1-p)^r - rp(1-p)^{r-1} \tag{1}$$

and

$$g''(p) = r\left[(r+1)p - 2\right](1-p)^{r-2}. \tag{2}$$

Lemma 1.
1. For $p \in [0,1]$ we have $g'(p) \geq 0$ if and only if $p \in \left[0, \frac{1}{r+1}\right]$.
2. The function g' is strictly decreasing for $p \in \left[0, \frac{2}{r+1}\right)$ and strictly increasing for $p \in \left(\frac{2}{r+1}, 1\right)$.
3. The function g' is nonincreasing for $p \in \left[0, \frac{2}{r+1}\right]$.

The information in this Lemma is summarized in Table 1.

Proof. The first part follows from the fact that $g'(p) \geq 0$ holds if and only if $(1-p)^r \geq rp(1-p)^{r-1}$, which holds if and only if $p \in \left[0, \frac{1}{r+1}\right]$. For the second part we need to characterize when $g''(p)$ is positive and when it is negative. Since, for $p \in [0,1)$, $r(1-p)^{r-2} > 0$, it follows that $g'(p)$ is strictly decreasing when $\left[(r+1)p-2\right] < 0$, which holds if and only if $p \in \left[0, \frac{2}{r+1}\right)$. Similarly it is strictly increasing if and only if $\left[(r+1)p-2\right] > 0$, which holds when $p \in \left(\frac{2}{r+1}, 1\right)$. The proof of the third part is similar to that of the second part. □

Proof of Proposition 1

Proof. A differentiable trace-form entropy satisfies the principle of transfers so long as $g'(p)$ is decreasing (Patil and Taillie, 1982, Theorem 4.2, with a typo: read $V'(\pi_j) \geq V'(\pi_i)$). From here the result follows by Lemma 1. □

Gradient

Generalized Simpson's entropy is given by

$$\zeta_r = \sum_{s=1}^{S} p_s(1-p_s)^r, \quad r = 1, 2, \ldots. \tag{3}$$

Since $\sum_{s=1}^{S} p_s = 1$, it can be written as a function of all probabilities but the last as

$$f(p_1, p_2, \ldots, p_{S-1}) = \sum_{s=1}^{S-1} p_s(1-p_s)^r + \left(1 - \sum_{s=1}^{S-1} p_s\right)\left(\sum_{s=1}^{S-1} p_s\right)^r. \tag{4}$$

The gradient of f is the vector $\left(\frac{\partial f}{\partial p_1}, \frac{\partial f}{\partial p_2}, \ldots, \frac{\partial f}{\partial p_{S-1}}\right)$, where for $u = 1, 2, \ldots, (S-1)$

$$\frac{\partial f}{\partial p_u} = (1-p_u)^r - p_u r(1-p_u)^{r-1} - \left(\sum_{s=1}^{S-1} p_s\right)^r + \left(1 - \sum_{s=1}^{S-1} p_s\right) r \left(\sum_{s=1}^{S-1} p_s\right)^{r-1}. \tag{5}$$

Table 1. Variation table of the function g

p	0	$\frac{1}{S}$	$\frac{1}{r+1}$	$\frac{2}{r+1}$		1
$g''(p)$		$-$		0	$+$	
$g'(p)$	1 ↘	↘	0 ↘	$-\left(\frac{r-1}{r+1}\right)^{r-1}$	↗	0

Hessian

The Hessian of f is the $(S-1)\times(S-1)$ matrix with $\frac{\partial^2 f}{\partial p_v \partial p_u}$ in position (u,v), where for $v \neq u$

$$\frac{\partial^2 f}{\partial p_v \partial p_u} = -2r\left(\sum_{s=1}^{S-1} p_s\right)^{r-1} + \left(1-\sum_{s=1}^{S-1} p_s\right) r(r-1)\left(\sum_{s=1}^{S-1} p_s\right)^{r-2} \tag{6}$$

and

$$\frac{\partial^2 f}{\partial p_u^2} = -2r(1-p_u)^{r-1} + p_u r(r-1)(1-p_s)^{r-2} - 2r\left(\sum_{s=1}^{S-1} p_s\right)^{r-1} + \left(1-\sum_{s=1}^{S-1} p_s\right) r(r-1)\left(\sum_{s=1}^{S-1} p_s\right)^{r-2}. \tag{7}$$

Extremum when all probabilities are equal

Proposition 1. *When all probabilities are equal, the generalized Simpson's entropy reaches a local maximum if $r+1 < 2S$ and a local minimum if $r+1 > 2S$.*

Proof. When $p_s = \frac{1}{S}$ for each $s = 1,2,\dots,S$

$$\frac{\partial f}{\partial p_s} = \left(\frac{S-1}{S}\right)^r - \frac{1}{S} r\left(\frac{S-1}{S}\right)^{r-1} - \left(\frac{S-1}{S}\right)^r + \frac{1}{S} r\left(\frac{S-1}{S}\right)^{r-1} = 0, \tag{8}$$

which means that the gradient is zero and this is a critical point. At this point the Hessian contains terms

$$\frac{\partial^2 f}{\partial p_u^2} = \frac{r}{S}\left(\frac{S-1}{S}\right)^{r-2} 2\left[r-2S+1\right] \tag{9}$$

and for $v \neq u$

$$\frac{\partial^2 f}{\partial p_v \partial p_u} = -2r\left(\frac{S-1}{S}\right)^{r-1} + \left(\frac{1}{S}\right) r(r-1)\left(\frac{S-1}{S}\right)^{r-2} = \frac{r}{S}\left(\frac{S-1}{S}\right)^{r-2}\left[r-2S+1\right]. \tag{10}$$

Denote $h(S,r) = \frac{r}{S}\left(\frac{S-1}{S}\right)^{r-2} 2\left[r-2S+1\right]$. The Hessian matrix is

$$\mathbf{H} = h(S,r)\begin{pmatrix} 2 & 1 & \cdots & 1 \\ 1 & \ddots & \ddots & \vdots \\ \vdots & \ddots & \ddots & 1 \\ 1 & \cdots & 1 & 2 \end{pmatrix}. \tag{11}$$

It is easy to check that the matrx $\mathbf{H}/h(S,r)$ is positive definite. Thus $\mathbf{H}$ is positive definite if $h(S,r) > 0$ and negative definite if $h(S,r) < 0$. The sign of $h(S,r)$ is that of $r-2S+1$. Thus, by the second derivative test, when all probabilities are equal

- f reaches a local maximum if $r+1<2S$;
- f reaches a local minimum if $r+1>2S$.

This completes the proof. □

Maximum of the function

The proof of Proposition 2 follows immediately from the following.

Proposition 2. *Let $r \le S-1$.*
1. The global maximum of ζ_r is reached when all proportions are equal.
2. There are no other local maxima.

Proof. From (5) it follows that

$$\frac{\partial f}{\partial p_u} = g'(p_u) - g'\left(1-\sum_{s=1}^{S-1} p_s\right). \tag{12}$$

Thus, when the gradient equals zero it means that, for every $u=1,2,\dots,(S-1)$, we have

$$g'(p_u) = g'\left(1-\sum_{s=1}^{S-1} p_s\right),$$

which implies that

$$g'(p_1) = g'(p_2) = \dots = g'(p_S). \tag{13}$$

To guarantee that $\sum_{s=1}^{S} p_s = 1$ there must be at least one $u \in \{1,2,\dots,S\}$ with $p_u \le \frac{1}{S}$. The assumption that $r \le (S-1)$ implies that $p_u \le \frac{1}{r+1}$. Combining this with Lemma 1 implies that $g'(p_u) \ge 0$. Combining this with (13) implies that $g'(p_s) \ge 0$ for each s. By Lemma 1 this means that $p_s \le \frac{1}{r+1}$ for each s. Since, by Lemma 1, g' is strictly decreasing on $\left(0, \frac{2}{r+1}\right)$ it follows that $p_1 = p_2 = \dots = p_S = 1/S$. Thus the only critical point is at the uniform distribution. By Proposition 1 this is a local maximum, hence it is the global maximum as well. □

Remark 1. *In summary, Propositions 1 and 2 imply that*

- *When $r \le S-1$, ζ_r has a global maximum at the uniform distribution.*
- *When $S \le r \le 2S-1$, ζ_r has a local maximum at the uniform distribution.*
- *When $r \ge 2S$, ζ_r has a local minimum at the uniform distribution.*

This leaves the question of whether, in the case $S \le r \le 2S-1$, the local maximum at the uniform distribution needs to be a global maximum. In general it does not. To illustrate this we consider the simple case where $p_1 = p$ and $p_u = \frac{1-p}{S-1}$ for $u = 2,3,\dots,S$. In this case

$$\zeta_r = u(p) = p(1-p)^r + (1-p)\left(1-\frac{1-p}{S-1}\right)^r.$$

In Figure 1 a plot of u is given for $S=10$ and $r=15 \in [S, 2S-1]$. The plot show that, in this case, the global maximum is not at the uniform distribution.

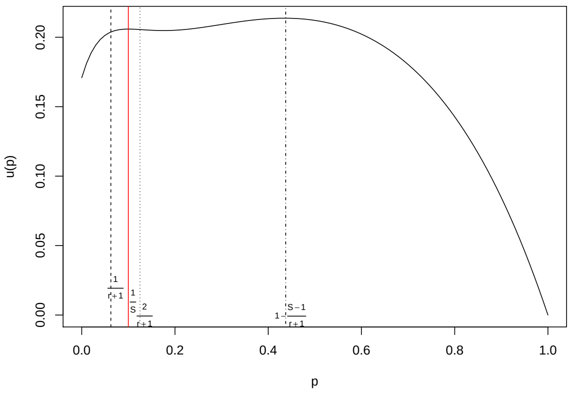

Figure 1. Plot of $u(p)$ for $S = 10$ and $r = 15$

Confidence intervals

The confidence intervals follow immediately from the following result.

Proposition 3. *If $r \leq S - 1$ and there exists an s with $p_s \neq 1/S$ then*

$$\sqrt{n}\frac{Z_v - \zeta_v}{\hat{\sigma}_v} \xrightarrow{L} N(0,1) \text{ as } n \to \infty. \tag{14}$$

Proof. In Zhang and Grabchak (2016) it was shown that (14) holds for any r and any $(p_1, p_2, \ldots, p_S)$ for which the gradient is not zero. Proposition 1 and the proof of Proposition 2 imply that this always holds under the given conditions. □

References

Patil GP, Taillie C (1982) Diversity as a concept and its measurement. Journal of the American Statistical Association 77(379):548–561

Zhang Z, Grabchak M (2016) Entropic Representation and Estimation of Diversity Indices. Journal of Nonparametric Statistics 28(3):563–575
