# Supplementary material for: The generalized Simpson’s entropy is a measure of biodiversity
Source: PLoS One. 2017 Mar 7;12(3):e0173305. doi: 10.1371/journal.pone.0173305 (PMC5340404; doi:10.1371/journal.pone.0173305)
Supplement: S2 Appendix — This code allows for the reproduction of all examples and figures in this article. (PDF) [file pone.0173305.s002.pdf]

The Generalized Simpson's Entropy is a Measure of Biodiversity. *PLOS ONE*.
Grabchak, M., Marcon, É., Lang, G. & Zhang, Z.
Corresponding author: Éric Marcon, UMR EcoFoG. (Eric.Marcon@ecofog.gf)

S2 Appendix: R code

```
library("entropart")
library("EntropyEstimation")

############## General settings ########################
# Generalized Simpson function
q.seq <- c(seq(0, .1, .025), seq(.15, .65, .05), seq(.7, 2, .1))
NumberOfSimulations <- 1000
Alpha <- 0.05
########################################################



############## Plot P6 and P18, data ########################
# Get data
data(Paracou618)
NsP6 <- as.AbdVector(Paracou618.MC$Nsi[, 1])
NsP18 <- as.AbdVector(Paracou618.MC$Nsi[, 2])
# Richness
(Richness(NsP6, Correction = "None"))
(S6 <- Richness(NsP6, Correction="Jackknife"))
(Richness(NsP18, Correction = "None"))
(S18 <- Richness(NsP18, Correction="Jackknife"))
S <- min(S6, S18)



############## Plot P6 and P18, HCDT ########################
# Calculate HCDT diversity profiles
D6 <- CommunityProfile(Diversity, NsP6, NumberOfSimulations=NumberOfSimulations,
        q.seq=q.seq, Correction="UnveilJ")
D18 <- CommunityProfile(Diversity, NsP18, NumberOfSimulations=NumberOfSimulations,
        q.seq=q.seq, Correction="UnveilJ")

# Plot both profiles
plot(D18$x, D18$y, ylim=c(min(D18$low)*.9, max(D18$high)*1.05), main="",
     xlab = "Order of Diversity", ylab = "Diversity", xlim=range(q.seq), type="n")
CEnvelope(D6, LineWidth=2, main="", xlim=range(q.seq), ShadeColor=NA, col="darkgreen")
lines(D6$x, D6$high, col="darkgreen", lty=1)
lines(D6$x, D6$low, col="darkgreen", lty=1)
CEnvelope(D18, lty=2, LineWidth=2, col="red", BorderColor="red", ShadeColor="NA")


############## Plot P6 and P18, zeta ########################
# Gen Simpson profile, plot 6
zeta6 <- CommunityProfile(GenSimpson, NsP6, 1:(S-1))
sigma6 <- sapply(1:(S-1), function(r) GenSimp.sd(NsP6,r))
ic6 <- qnorm(1-Alpha/2)*sigma6/sqrt(sum(NsP6))
zeta6$low <- zeta6$y - ic6
zeta6$high <- zeta6$y + ic6
```

```
# Gen Simpson profile, plot 18
zeta18 <- CommunityProfile(GenSimpson, NsP18, 1:(S-1))
sigma18 <- sapply(1:(S-1), function(r) GenSimp.sd(NsP18,r))
ic18 <- qnorm(1-Alpha/2)*sigma18/sqrt(sum(NsP18))
zeta18$low <- zeta18$y - ic18
zeta18$high <- zeta18$y + ic18

# Plot both profiles
plot(zeta18$x, zeta18$y, ylim=c(min(zeta6$low)*.9, 1), main="", xlab = "Order of Diversity",
     ylab = "Generalized Simpson Entropy", xlim=c(1, S-1), type="n")
CEnvelope(zeta6, LineWidth=2, main="", xlim=c(1, S-1), ShadeColor=NA, col="darkgreen")
lines(zeta6$x, zeta6$high, col="darkgreen", lty=1)
lines(zeta6$x, zeta6$low, col="darkgreen", lty=1)
CEnvelope(zeta18, lty=2, LineWidth=2, col="red", BorderColor="red", ShadeColor="NA")


############## Plot P6 and P18, zeta Diversity ###########
# Transform entropy into diversity
zeta6D <- zeta6
zeta6D$y <- 1/(1-(zeta6$y)^(1/zeta6$x))
zeta6D$low <- 1/(1-(zeta6$low)^(1/zeta6$x))
zeta6D$high <- 1/(1-(zeta6$high)^(1/zeta6$x))
zeta18D <- zeta18
zeta18D$y <- 1/(1-(zeta18$y)^(1/zeta18$x))
zeta18D$low <- 1/(1-(zeta18$low)^(1/zeta18$x))
zeta18D$high <- 1/(1-(zeta18$high)^(1/zeta18$x))
# Plot both profiles
plot(zeta18D$x, zeta18D$y, ylim=c(min(zeta6D$low)*.9, max(zeta18D$high)*1.05), main="",
     xlab = "Order of Diversity", ylab = "Generalized Simpson Diversity",
     xlim=c(1, S-1), type="n")
CEnvelope(zeta6D, LineWidth=2, main="", xlim=c(1, S-1), ShadeColor=NA, col="darkgreen")
lines(zeta6D$x, zeta6D$high, col="darkgreen", lty=1)
lines(zeta6D$x, zeta6D$low, col="darkgreen", lty=1)
CEnvelope(zeta18D, lty=2, LineWidth=2, col="red", BorderColor="red", ShadeColor="NA")


############## Plot difference of entropy #######################
# Calculate P18-P6 with CI
Difference <- list(x=zeta18$x, y=zeta18$y-zeta6$y)
class(Difference) <- class(zeta18)
icDifference <- qnorm(1-Alpha/2)*sqrt(sigma6^2/sum(NsP6) + sigma18^2/sum(NsP18))
Difference$low <- Difference$y - icDifference
Difference$high <- Difference$y + icDifference
# Plot
plot(Difference, ylab="Difference of entropy")
abline(h=0, col="blue", lty=2)


############## Hurlbert #######################
# Hurlbert
N6 <- sum(NsP6)
N18 <- sum(NsP18)
N <- min(N6, N18)
Dk6 <- CommunityProfile(Hurlbert, NsP6, NumberOfSimulations=NumberOfSimulations, q.seq=2:N)
Dk18 <- CommunityProfile(Hurlbert, NsP18, NumberOfSimulations=NumberOfSimulations, q.seq=2:N)
```

```
# Plot both profiles
plot(Dk18$x, Dk18$y, ylim=c(min(Dk18$low)*.9, max(Dk18$high)*1.05), main="",
     xlab = "Order of Diversity", ylab = "Hurlbert Diversity", xlim=c(2,N), type="n")
CEnvelope(Dk6, LineWidth=2, main="", xlim=c(2,N), ShadeColor=NA, col="darkgreen")
lines(Dk6$x, Dk6$high, col="darkgreen", lty=1)
lines(Dk6$x, Dk6$low, col="darkgreen", lty=1)
CEnvelope(Dk18, lty=2, LineWidth=2, col="red", BorderColor="red", ShadeColor="NA")


############## Equivalence of orders ###########
# Min q value at max r
(qMin6 <- D6$x[which.min(D6$y > max(zeta6D$y))])
qOK6 <- which(D6$y < max(zeta6D$y))
(qMin18 <- D18$x[which.min(D18$y > max(zeta18D$y))])
qOK18 <- which(D18$y < max(zeta18D$y))

# Extend P18 zeta
zeta18e <- CommunityProfile(GenSimpson, NsP18, 1:(S18-1))
sigma18e <- sapply(1:(S18-1), function(r) GenSimp.sd(NsP18,r))
ic18e <- qnorm(1-Alpha/2)*sigma18e/sqrt(sum(NsP18))
zeta18e$low <- zeta18e$y - ic18e
zeta18e$high <- zeta18e$y + ic18e
# diversity
zeta18eD <- zeta18e
zeta18eD$y <- 1/(1-(zeta18e$y)^(1/zeta18e$x))
zeta18eD$low <- 1/(1-(zeta18e$low)^(1/zeta18e$x))
zeta18eD$high <- 1/(1-(zeta18e$high)^(1/zeta18e$x))
plot(zeta18eD)
# Min q value at max r
(qMin18e <- D18$x[which.min(D18$y > max(zeta18eD$y))])
qOK18e <- which(D18$y < max(zeta18eD$y))



############## Appendix proofs ########################
# univariate function u
u <- function(p, S, r) {
  return( (1-p)*(1-(1-p)/(S-1))^r + p*(1-p)^r )
}
# graphical parameters
rshift <- .01
tcex <- .7
# parameters
S <- 10
r <- 15
curve(u(x, S=S, r=r), from=0, to=1, xlab="p", ylab="u(p)")
abline(v=1/S, col="red")
text(x = 1/S +rshift, y = 0.01, labels=expression(frac(1,S)), cex=tcex)
abline(v=1/(r+1), lty=2)
text(x = 1/(r+1) +rshift, y = 0.02, labels=expression(frac(1,r+1)), cex=tcex)
abline(v=2/(r+1), lty=3)
text(x = 2/(r+1) +rshift, y = 0, labels=expression(frac(2,r+1)), cex=tcex)
abline(v=1-(S-1)/(r+1), lty=4)
text(x = 1-(S-1)/(r+1) +rshift, y = 0, labels=expression(1-frac(S-1,r+1)), cex=tcex)
```
